# Systematic review of emergency medicine clinical practice guidelines: Implications for research and policy

**DOI:** 10.1371/journal.pone.0178456

**Published:** 2017-06-19

**Authors:** Arjun K. Venkatesh, Dan Savage, Benjamin Sandefur, Kenneth R. Bernard, Craig Rothenberg, Jeremiah D. Schuur

**Affiliations:** 1 Department of Emergency Medicine, Yale University School of Medicine, New Haven, CT, United States of America; 2 Yale New Haven Hospital Center for Outcomes Research and Evaluation, New Haven, CT, United States of America; 3 Department of Emergency Medicine, Mayo Clinic, Rochester, MN, United States of America; 4 Brigham and Women’s Hospital-Massachusetts General Hospital-Harvard Affiliated Emergency Medicine Residency, Boston, MA, United States of America; 5 Department of Emergency Medicine, Brigham and Women’s Hospital, Boston, MA, United States of America; University of Liverpool, UNITED KINGDOM

## Abstract

**Introduction:**

Over 25 years, emergency medicine in the United States has amassed a large evidence base that has been systematically assessed and interpreted through ACEP Clinical Policies. While not previously studied in emergency medicine, prior work has shown that nearly half of all recommendations in medical specialty practice guidelines may be based on limited or inconclusive evidence. We sought to describe the proportion of clinical practice guideline recommendations in Emergency Medicine that are based upon expert opinion and low level evidence.

**Methods:**

Systematic review of clinical practice guidelines (Clinical Policies) published by the American College of Emergency Physicians from January 1990 to January 2016. Standardized data were abstracted from each Clinical Policy including the number and level of recommendations as well as the reported class of evidence. Primary outcomes were the proportion of Level C equivalent recommendations and Class III equivalent evidence. The primary analysis was limited to current Clinical Policies, while secondary analysis included all Clinical Policies.

**Results:**

A total of 54 Clinical Policies including 421 recommendations and 2801 cited references, with an average of 7.8 recommendations and 52 references per guideline were included. Of 19 current Clinical Policies, 13 of 141 (9.2%) recommendations were Level A, 57 (40.4%) Level B, and 71 (50.4%) Level C. Of 845 references in current Clinical Policies, 67 (7.9%) were Class I, 272 (32.3%) Class II, and 506 (59.9%) Class III equivalent. Among all Clinical Policies, 200 (47.5%) recommendations were Level C equivalent, and 1371 (48.9%) of references were Class III equivalent.

**Conclusions:**

Emergency medicine clinical practice guidelines are largely based on lower classes of evidence and a majority of recommendations are expert opinion based. Emergency medicine appears to suffer from an evidence gap that should be prioritized in the national research agenda and considered by policymakers prior to developing future quality standards.

## Introduction

Over the last fifty years medicine has increasingly moved away from anecdotal replication of practice patterns taught during training and embraced evidence-based medicine. This transition is most strongly embodied by the widespread publication and dissemination of clinical practice guidelines by medical specialty societies, health care institutions, and governmental bodies.[1] Such guidelines aim to advance the quality of healthcare delivery by summarizing the best available evidence in order to accelerate knowledge translation and reduce variations in practice.[2, 3]

The first Emergency medicine clinical practice guidelines were developed by the American College of Emergency Physicians (ACEP) in 1990 for the management of patients with chest pain.[4] While emergency medicine clinical practice guidelines, which have primarily been authored by the American College of Emergency Physicians (ACEP) as ACEP Clinical Policies, have generated substantial discussion and debate over the years [5], these clinical policies serve as the foundation of educational programs, the development of quality measures and as the basis for research and advocacy efforts.

While the development process used for clinical practice guideline development is designed to reflect the highest quality of available evidence for clinical scenarios, prior evaluations of clinical guidelines in cardiology, obstetrics and gynecology and infectious disease found that many recommendations are based on lower-classes of evidence quality with the majority of recommendations based on expert opinion and low level evidence[6–8]. ACEP Clinical Policies have been designed to answer critical clinical questions in emergency medicine based on the systematic appraisal and interpretation of available evidence by an expert group. Often, however, Clinical Policies may be based on limited or inconclusive evidence. For example, the ACEP Clinical Policy recommending thrombolytics for hemodynamically unstable patients with pulmonary embolism or the recommendation that “pain response should not be used as the sole diagnostic indicator of the underlying etiology of an acute headache” [9] reflect this phenomena, respectively. Identifying gaps in clinical evidence underlying Clinical Policy recommendations is needed to define the future research agenda and inform policymakers seeking to develop measures of emergency care quality. To date, despite attempts to strengthen the ACEP Clinical Policy development process and growth in emergency care research funding and output, the strength of evidence that supports recommendations within emergency medicine promulgated clinical guidelines is not known.[10–13]

Accordingly, we sought to describe the proportion of Clinical Policy recommendations that are based upon expert opinion and low level evidence, and to describe the classes of evidence supporting these ACEP Clinical Policies. We also sought to examine trends in the emergency care evidence base by describing trends in both recommendations and evidence classification.

## Methods

### Study design

Systematic review of American College of Emergency Physicians clinical policies using PRISMA guidelines (S1 Appendix).

### Selection of clinical practice guidelines

**Fig 1:**
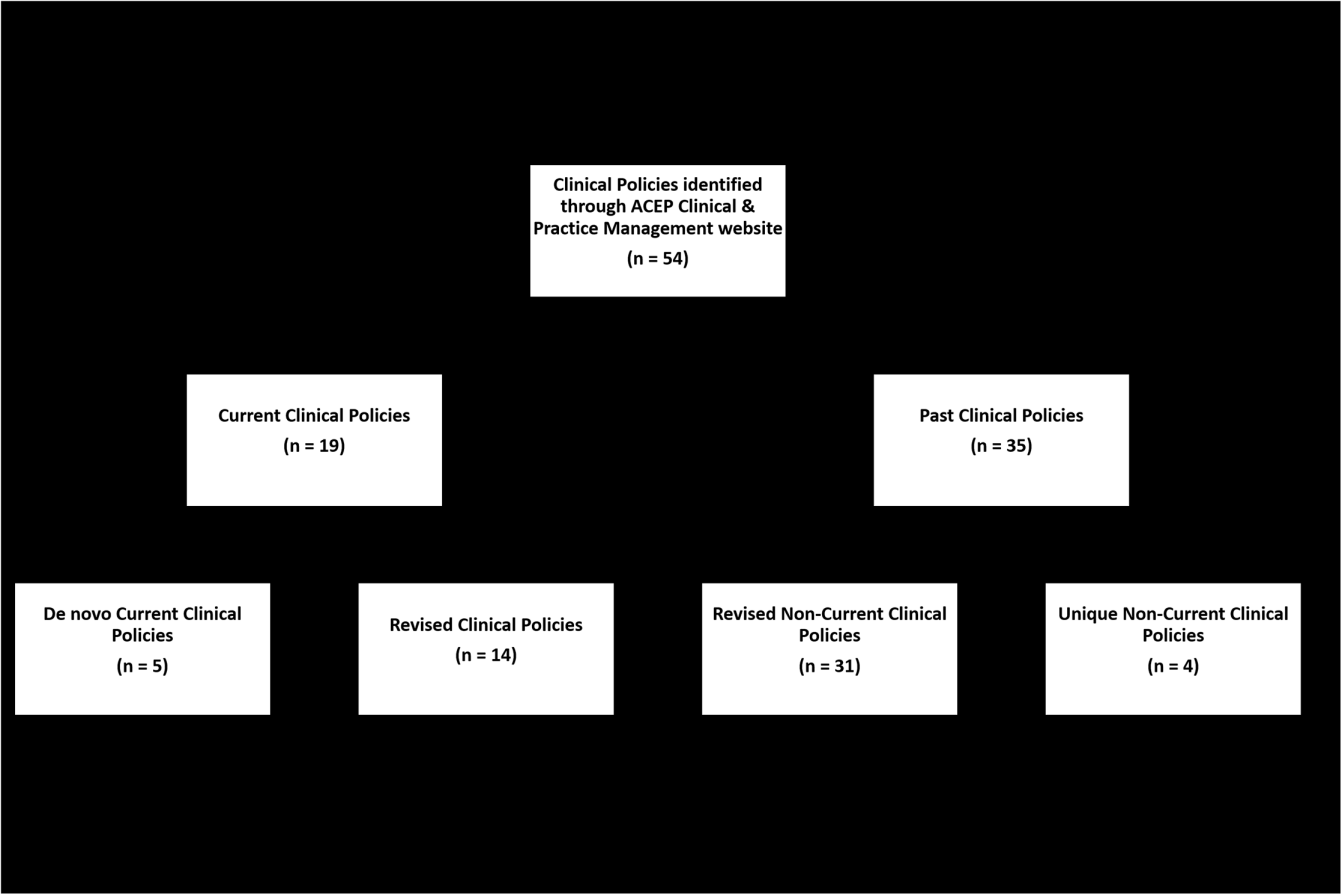
PRISMA diagram for systematic review of clinical policies.

We included all American College of Emergency Physician Clinical Policies listed as both “current” and “past” from the ACEP Clinical & Practice Management website, http://www.acep.org/clinicalpolicies/. ACEP Clinical Policies are the only regularly published, medical specialty society sponsored clinical practice guidelines specific to emergency care in the United States. Each Clinical Policy is published as a peer-reviewed manuscript containing evidence-based recommendations designed to guide clinical practice. A Clinical Policy was defined as “current” if it was listed as such and available for download on the ACEP Clinical & Practice Management website as of October 20, 2015. A Clinical Policy was defined as “non-current” if it was listed as “past” on the ACEP Clinical & Practice Management website. We did not include clinical guidelines published by other professional organizations in emergency medicine, as they have neither a regular process nor a group charged with regular clinical guideline development and maintenance. We also did not include clinical guidelines published primarily by other organizations and co-signed or endorsed by ACEP as they followed a different review and writing process (**Fig 1).**

### Data definitions

Each Clinical Policy was reviewed for three types of data elements using a standardized data collection tool in Microsoft Excel (Microsoft Corp., 2007, Redmond, WA): bibliographic; evidence-based; and recommendation-based data elements.

The bibliographic data collected included date published, the clinical category focus of the guideline (chief-complaint; disease-specific; procedure/intervention), current or non-current Clinical Policy status, whether it is a revision of the prior Clinical Policy, and whether other clinical societies (e.g. the American College of Cardiology) were involved in authorship.

The evidence-based data elements were abstracted from the bibliography of each Clinical Policy in which each reference is graded as Class I, II, or III (**S1 Table).** Each Clinical Policy includes a compendium of references in a single evidentiary table, so evidence grading can only be abstracted at the Clinical Policy level and not specific to each recommendation. References in the bibliography or evidentiary table that were not assigned a class of evidence were excluded. This was particularly applicable to the oldest Clinical Policies because evidence was not always explicitly documented between 1990 to 1995 with the first comprehensive classification of evidence appearing in Clinical Policies after 1996 [14]. Because the exact definitions of Class I, Class II, and Class III evidence evolved minimally, we created a single definition to standardize comparison that included older Clinical Policies. Class I equivalent evidence for therapeutic interventions constitutes randomized controlled trials or meta-analysis of randomized controlled trials, while Class I equivalent evidence for diagnostic interventions or prognostication constitutes prospective cohort studies or meta-analyses of the same. Class II equivalent evidence reflects nonrandomized trials for therapeutic interventions, or retrospective studies for diagnostic or prognostic actions. Class III equivalent evidence consists of case series, case reports, expert consensus, or other reviews. The current ACEP Clinical Policy literature classification schema can be found in S1 Table.

Individual Clinical Policy recommendations are defined as the set of strategies for which “medical literature exists to provide support for answers to critical questions” [15] faced by physicians working in emergency departments. For each recommendation we abstracted the type of recommendation (therapeutic, diagnostic, prognostic, or procedural), the level of strength of the recommendation (A, B, or C), and whether the recommendation was focused on adult or pediatric patients. In 1998, the scoring rubric for the three-tiered strength of recommendation was changed. To standardize the scoring and tabulation of strength of recommendations across all Clinical Policies, a single definition was used that aligned closely with current Clinical Policies. All Clinical Policies included in the primary analyses were rated using the current definitions without modification. S1 Text. Level A equivalent recommendations are those generally accepted principles for patient management that reflect a high degree of clinical certainty, meaning that they are based on Class I strength of evidence or overwhelming Class II evidence. Level B equivalent recommendations are those based on moderate clinical certainty, such as those based on Class II evidence including decision analysis that directly addresses the issue, or strong consensus of Class III studies. Level C equivalent recommendations were defined as those based on preliminary or inconclusive evidence, committee consensus, or limited-research-based evidence.

### Data abstraction

Data were abstracted by three authors (BS, KB, DS) from the first ACEP Clinical Policy in 1990 until 2009 with perfect inter-rater reliability for the extracted data, except for the levels of recommendation for one Clinical Policy [16], which is no longer current, in which no distinction was made between level B or C recommendations. Given this ambiguity, a consensus was reached to classify all three recommendations (0.7% of all recommendations reviewed) in this Clinical Policy as level C equivalent as they were based on 18 studies rated as class II evidence, and 33 studies rated as class III evidence. A single author (DS) completed the data extraction for all CPGs published between January 2010 and January 2015 without double extraction given near perfect agreement during the initial data review.

### Outcomes

The primary outcomes were the proportion of all current recommendations reported as level C equivalent and the proportion of all current evidence reported as class III equivalent.

#### Primary analysis

We report descriptive statistics including the mean number of recommendations and mean number of references included within each Clinical Policy. We also report each outcome by year and use the Cochrane Armitage test for trend to evaluate the change in the proportion of Level C Recommendations and Class III Equivalent evidence over time. We also use chi-square tests to describe differences in the primary outcome between current and non-current Clinical Policies.

#### Secondary analysis

Because many Clinical Policies provide recommendations for unique clinical indications are available in the literature or secondary clinical knowledge tools despite no longer being listed as “current” by ACEP, and because several earlier versions of Clinical Policies contain unique recommendations not included in future Clinical Policies, we also conducted a secondary analysis of all Clinical Policies in the dataset. For example, “Critical Issues in the Evaluation and Management of Adult Patients Presenting to the Emergency Department With Seizures” has been revised four times—2014[17], 2004[18], 1997[19], and 1993[20]. In the primary analysis, only the 2014 iteration is included in the analysis of current Clinical Policies. However, because the current 2014 Clinical Policy only addresses three of the six topics addressed in its 2004 version[18], all recommendations and graded evidence in the three prior versions are included in the secondary analysis of all Clinical Policies.

## Results

Between January 1990 and January 2016 ACEP published 54 Clinical Policies at a mean rate of 2.3 Clinical Policies per year (range 0–4, median 2). These Clinical Policies cover 27 unique topics of which 9 have no published revision or are first version Clinical Policies and 45 are updates or expansions upon prior Clinical Policies. A total of 25 Clinical Policies are clinically focused on a chief complaint (e.g. headache), 21 on a specific disease (e.g. appendicitis), and 8 on a procedure or intervention (e.g. procedural sedation and analgesia).

Of all Clinical Policies, 19 (35%) were classified as current Clinical Policies, the oldest of which was published in 2003.[21] Policies were primarily authored by ACEP with several guidelines collaboratively developed with other specialty societies (14.3%).

Among the 19 current ACEP Clinical Policies, 14 were updates or revisions of prior guidelines and 5 represented the first version on a given topic. These current Clinical Policies contain 141 recommendations (range 2–13 per guideline, mean 7.4), of which 13 were Level A equivalent recommendations (9.2%), 57 Level B equivalent recommendations (40.4%), and 71 Level C Equivalent Recommendations (50.4%). In total the current clinical policies contained 845 graded pieces of evidence (mean 44.5, range: 6–119 per Clinical Policy). Sixty-seven pieces of evidence were Class I equivalent (7.9%), 272 were Class II equivalent (32.2%), and 506 were Class III Equivalent (59.9%).

Among the 14 current Clinical Policies that are the most recent revisions of 23 prior Clinical Policies, the proportion of level C equivalent recommendations increased from 36.3% in prior iterations to 44.7% in the current 14 Clinical Policies. Similarly, the proportion of Class III equivalent evidence also increased from 42.3% to 60.0%.

Of 421 total recommendations included in all 54 Clinical Policies in the dataset, 37 (8.8%) were Level A equivalent recommendations, 184 (43.7%) Level B equivalent recommendations, and 200 (47.5%) Level C equivalent recommendations.

**Fig 2:**
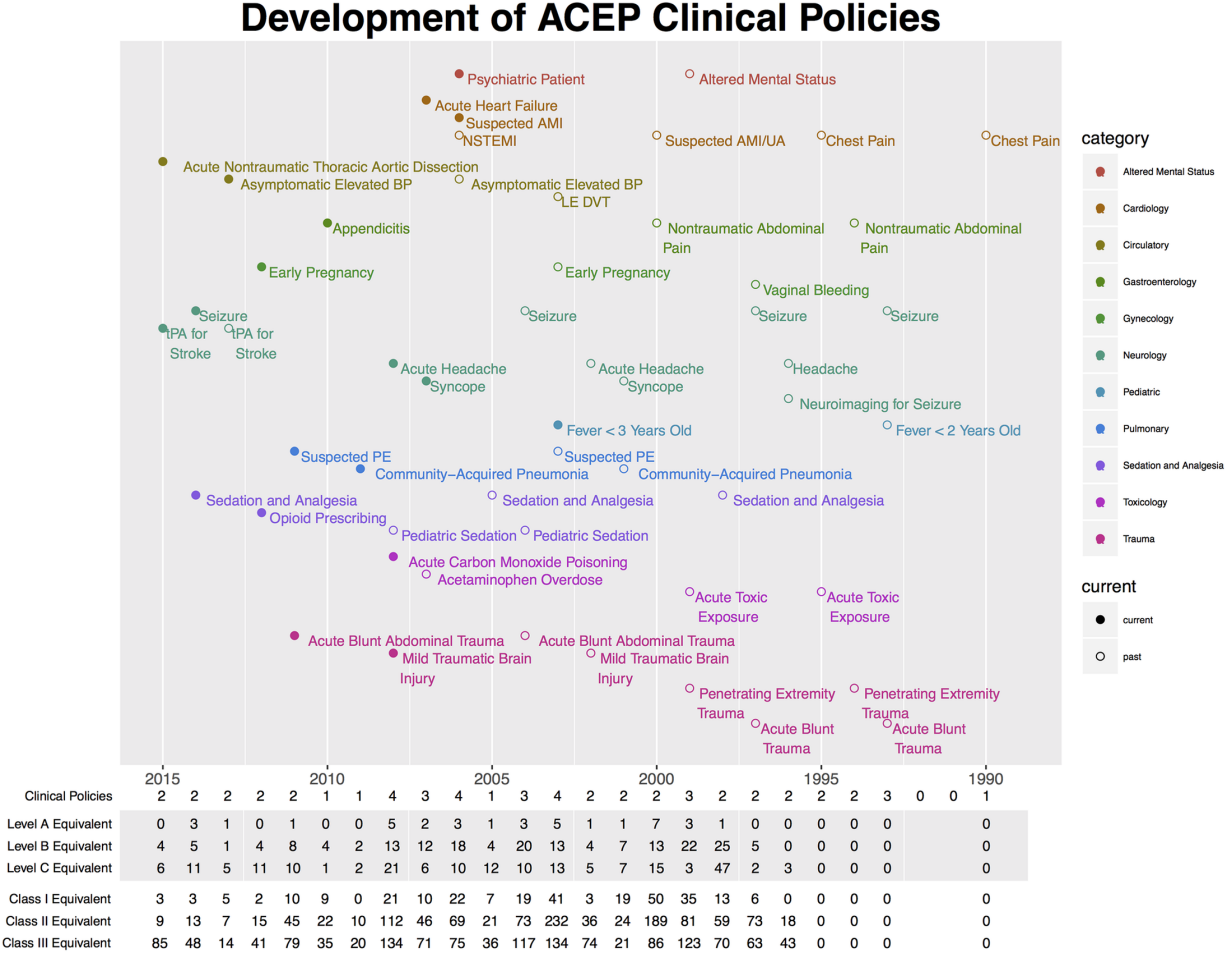
Development timeline of clinical policies.

The proportion of Level C Equivalent recommendations in current Clinical Policies was not significantly different than non-current Clinical Policies (47.2% vs 48.4%, p-value = 0.82) (**Table 1).** There was no statistically significant trend in the proportion of Level C recommendations in guidelines between 1996, the first year of structured grading of recommendations, and January 2016 (two-sided Cochrane Armitage trend test p = 0.30) (**Table 2 and Fig 2).**

**Table 1:** Distribution of recommendation level and evidence class in current and non-current clinical policies.

	Current	Non-Current
**Recommendations**		
** Level A**	13 (11%)	24 (8%)
** Level B**	50 (41%)	134 (45%)
** Level C**	59 (48%)	141 (47%)
** Total**	122	299
**Evidence cited**		
** Class I**	67 (8%)	211 (11%)
** Class II**	272 (32%)	882 (45%)
** Class III**	504 (60%)	865 (44%)
** Total**	843	1958

**Table 2:** Distribution of recommendation levels and evidence class by year.

Year*		2015	2014	2013	2012	2011	2010	2009	2008	2007	2006	2005	2004	2003	2002	2001	2000	1999	1998
Total Recommendations		10	19	7	15	19	5	4	39	20	31	17	33	31	10	15	35	28	73
	Level A	0 (0%)	3 (16%)	1 (14%)	0 (0%)	1 (5%)	0 (0%)	0 (0%)	5 (13%)	2 (10%)	3 (10%)	1 (6%)	3 (9%)	5 (16%)	1 (10%)	1 (7%)	7 (20%)	3 (11%)	1 (1%)
	Level B	4 (40%)	5 (26%)	1 (14%)	4 (27%)	8 (42%)	4 (80%)	2 (50%)	13 (33%)	12 (60%)	18 (58%)	4 (24%)	20 (61%)	13 (42%)	4 (40%)	7 (47%)	13 (37%)	22 (79%)	25 (34%)
	Level C	6 (60%)	11 (58%)	5 (71%)	11 (73%)	10 (53%)	1 (20%)	2 (50%)	21 (54%)	6 (30%)	10 (32%)	12 (71%)	10 (30%)	13 (42%)	5 (50%)	7 (47%)	15 (43%)	3 (11%)	47 (64%)
Total Evidence		97	64	26	58	134	66	30	267	127	166	64	209	407	113	64	325	239	142
	Class I	3 (3%)	3 (5%)	5 (19%)	2 (3%)	10 (7%)	9 (14%)	0 (0%)	21 (8%)	10 (8%)	22 (13%)	7 (11%)	19 (9%)	41 (10%)	3 (3%)	19 (30%)	50 (15%)	35 (15%)	13 (9%)
	Class II	9 (9%)	13 (20%)	7 (27%)	15 (26%)	45 (34%)	22 (33%)	10 (33%)	112 (42%)	46 (36%)	69 (42%)	21 (33%)	73 (35%)	232 (57%)	36 (32%)	24 (38%)	189 (58%)	81 (34%)	59 (42%)
	Class III	85 (88%)	48 (75%)	14 (54%)	41 (71%)	79 (59%)	35 (53%)	20 (67%)	134 (50%)	71 (56%)	75 (45%)	36 (56%)	117 (56%)	134 (33%)	74 (65%)	21 (33%)	86 (26%)	123 (51%)	70 (49%)

*Data prior to 1998 is excluded as the scoring rubric for recommendations was changed.

A total of 2801 references were graded across all clinical policies, of which 278 (9.9%) were Class I equivalent, 1154 (41.2%%) were Class II equivalent, and 1369 (48.9%) were Class III equivalent. The proportion of Class III Equivalent evidence contained in current clinical policies was significantly higher than non-current Clinical Policies (44.2% vs 59.8%, p-value = <0.0001). In addition, the proportion of Class III evidence in clinical practice guidelines significantly increased between 1996 and 2015 (two-sided Cochrane Armitage trend test: p<0.0001).

## Discussion

The development of clinical practice guidelines has become widespread across medical specialties including emergency medicine. We found that, like other specialties, close to half of current recommendations in emergency medicine clinical practice guidelines are based on expert opinion or lower classes of evidence rather than high quality clinical trials. We also found that clinical policies are increasingly likely to proportionally contain Level C recommendations and Class III evidence, including the subset of clinical policies that reflect updates and revisions to prior clinical policies.

These evidence gaps in current emergency care clinical practice guidelines reflect the design of the ACEP Clinical Policy development and reporting process as well as gaps in the emergency medicine evidence base. Some medical specialties develop clinical practice guidelines based on the existing evidence base in a manner consistent with several international consensus standards for clinical practice guideline development.[22] ACEP Clinical Polices, however, are developed based on a unique process: the policies answer a set of critical questions that are thought to be of key clinical importance and are defined prior to evidence review. As such, critical questions may not cover areas where there is definitive evidence, requiring consensus from content experts to address research equipoise. Such expert consensus may be of particular value when clinical recommendations are made for vulnerable populations such as the frail elderly or pregnant women that may be commonly excluded from clinical trials. Conversely, expert consensus may risk both codifying, or prolonging, anecdotal clinical practices that are based on limited evidence through the authority of a clinical practice guideline. In fact, on occasion prior work has shown that expert opinion may result in recommendations that depart from accepted practice and even recommend potentially harmful treatments despite contrary clinical evidence. [23, 24] The Clinical Policy reporting process could be improved to accommodate this need by developing a new, distinct recommendation Level for expert opinion recommendations that is distinct from the Level C used for lower quality empiric investigation as is proposed by the Oxford Center for Evidence Based Medicine and by the Institute of Medicine.[25, 26] A distinct designation for expert consensus recommendations would also permit the preliminary establishment of clinical standards and guidance prior to the publication of more definitive, higher classification research in the future.

A growing proportion of emergency care clinical practice guidelines are based on lower classes of evidence despite a growing body of emergency care research and publications. This evidence deficit may be permissible for recommendations that are unlikely to warrant the conduct of a randomized clinical trial such as the recommendation to obtain a blood glucose in seizure patients, which is consistent with good clinical practice but unlikely to ever meet the Level A threshold. On the other hand, clinical recommendations such as the use of both a parenteral benzodiazepine and haloperidol to produce more rapid sedation than monotherapy in the acutely agitated patient [27] or the proper timing to start an angiotensin-converting enzyme inhibitor in the initial management of heart failure [28] are well defined clinical scenarios that should be targets of controlled clinical trials worthy of Class I evidence designation. Acknowledging that Class I evidence to support all clinical practice in the emergency department is impractical, Federal agencies such as the National Institute of Health or the Agency for Healthcare Research and Quality should use critical appraisals of clinical practice guidelines such as this to identify topics of clinical importance and interest for which an evidence gap necessitates funding future investigation. Furthermore, researchers seeking to highlight the importance of research aims or to emphasize topics for investigation should look to gaps in Clinical Policies for such support.

In addition to research gaps, we also identified several opportunities to improve the quality of each Clinical Policy based on the recent recommendations of the IOM.[1] While our systematic review did not evaluate aspects of transparency, conflict of interest disclosure, writing group composite, or external review promoted by the IOM, our work did find that ACEP Clinical Policies broadly meet the rating and reporting guidance of the IOM. However, ACEP Clinical Policies, unlike other specialties societies such as the American College of Cardiology, do not meet the IOM recommendation to provide an evidence rating for each individual recommendation. The practice of only providing guideline-wide evidence summary tables may result in confusion as to the exact evidence base for a given recommendation—did a single observation study water down a recommendation in the face of limited prospective data, or did a single, small prospective clinical trial buoy up a recommendation? In addition, while fourteen of the 19 current Clinical Policies reflect updates or revision to prior Clinical Policies, we found that archived Clinical Policies contain many recommendations not subject to re-discussion or revision. As a result, some topics, or “Critical Questions,” still relevant to current practice may not undergo the regular re-evaluation recommended by the IOM. For example, in the Clinical Policies regarding adult patients with seizures, the 2004 version address the possible utility of various diagnostic evaluations such as serum glucose and sodium levels in patient with a first time seizure with no comorbidities who have returned to their baseline[18]; a topic no longer mentioned in the current 2014 Clinical Policy.[17] While embedding of prior recommendations in archived guidelines may be necessary due to the strict publication requirements of journals and specialty society reports, this practice may result in an underestimation of the strength of evidence that support several clinical practices and hinders the continued dissemination of important knowledge. Facing similar challenges as the speed and quantity of publications rapidly rises [29], the American Heart Association has developed a website that can be quickly updated to contain the most up to date recommendations. Such a process would result in clinical practice guidelines of higher relevance to practice and likely more clinician use at the bedside.[30, 31]

Our findings also carry several implications for policymakers. As formal criteria for the classes of evidence or requirements for clinical practice guidelines to support the endorsement or use of quality measures are adopted—the content and quality of Clinical Policies become of utmost importance for clinical standards used in new pay for performance programs[32–34] Policymakers must recognize the wide variability in the strength of evidence underlying Clinical Policies when selecting new targets for quality measurement as well as responding to quality measures developed outside of emergency medicine but with relevance to emergency care. For example, the ACEP Clinical Policy, *Critical issues in the evaluation and management of adult patients presenting to the emergency department with suspected pulmonary embolism*, includes one Level B recommendation and two Level C recommendations that serve as the basis for the quality measure, “Appropriate Emergency Department Utilization of CT for Pulmonary Embolism”included in the new CMS approved Clinical Emergency Department Registry (CEDR) (25).[35] In contrast, after CMS proposed a quality measure for the utilization of head CT for headache, the ACEP Clinical Policy, *Clinical Policy: Critical Issues In The Evaluation And Management Of Adult Patients Presenting To The Emergency Department With Acute Headache[9]* was cited in an evaluation of the measure that subsequently led to the withdrawal of the measure due to lack of supporting evidence. [36] A recent survey of the Guidelines International Network indicates that emergency medicine is not alone as a specialty seeking better integration of clinical guideline and quality measure development[37], particularly given the greater attention given to the methodology of clinical practice guidelines development in comparison to the use of evidence of quality and accountability measurement. Future efforts to develop metrics that closely mirror the narrow specifications of higher-level recommendations based on higher classes of evidence will ensure that quality measures do not result in “distortions to care,” or unintended consequences such as overtesting or the expansion of care processes to unintended populations.[38]

## Limitations

Several limitations of our work warrant mention. First, our systematic review was limited to clinical practice guidelines promulgated by the major emergency medicine specialty society in the United States and did not include the many clinical practice guidelines and recommendations for emergency care that may be contained within guidelines published by other specialty societies or in other countries. Second, the current design of ACEP Clinical Policies that published a summary evidence table precludes any assessment of the strength of evidence supporting individual recommendations—as such our conclusions reflect a broad assessment of current Clinical Policies as opposed to a detailed evaluation of individual clinical questions. Finally, our review abstracted recommendations and strength of designations as reported in each Clinical Policy and did not re-assess the strength of each recommendation or guideline using an independent scoring tool, such as AGREE II.[39] As such, our analysis better reflects current clinical practice guidelines as presented to readers, but detailed study of Clinical Policy component using established clinical practice guideline review tools may warrant future study.

## Conclusions

Nearly half of current emergency medicine clinical practice guideline recommendations are based on expert opinion and low level evidence rather than clinical trial evidence. Despite a rapidly expanding body of published emergency care research, clinical practice guidelines increasingly contain consensus-based recommendations based on lower classes of evidence. These evidence gaps in clinical guidelines highlight priorities for the future emergency care research agenda. Policymakers should be aware of the low quality of evidence behind many guideline recommendations when developing future quality standards.

## Supporting information

S1 Appendix PRISMA checklist. (DOC)

S1 Text Current ACEP definitions of strength of recommendations. (DOCX)

S1 Table ACEP literature classification schema. (DOCX)
