# Supplementary material for: Systematic review of emergency medicine clinical practice guidelines: Implications for research and policy
Source: PLoS One. 2017 Jun 19;12(6):e0178456. doi: 10.1371/journal.pone.0178456 (PMC5476239; doi:10.1371/journal.pone.0178456)
Supplement: S1 Text — (DOCX) [file pone.0178456.s002.docx]

**Supplemental Text 1. Current ACEP Definitions of Strength of Recommendations**

**Level A recommendations.** Generally accepted principles for patient care that reflect a high degree of clinical certainty (ie, based on evidence from 1 or more Class of Evidence I or multiple Class of Evidence II studies).

**Level B recommendations**. Recommendations for patient care that may identify a particular strategy or range of strategies that reflect moderate clinical certainty (ie, based on evidence from 1 or more Class of Evidence II studies or strong consensus of Class of Evidence III studies).

**Level C recommendations.** Recommendations for patient care that are based on evidence from Class of Evidence III studies or, in the absence of any adequate published literature, based on expert consensus. In instances where consensus recommendations are made, “consensus” is placed in parentheses at the end of the recommendation.
