# Supplementary figures and images for: Systematic review of emergency medicine clinical practice guidelines: Implications for research and policy
Source: PLoS One. 2017 Jun 19;12(6):e0178456. doi: 10.1371/journal.pone.0178456 (PMC5476239; doi:10.1371/journal.pone.0178456)

**Supplemental Table 1. ACEP Literature classification schema**

**
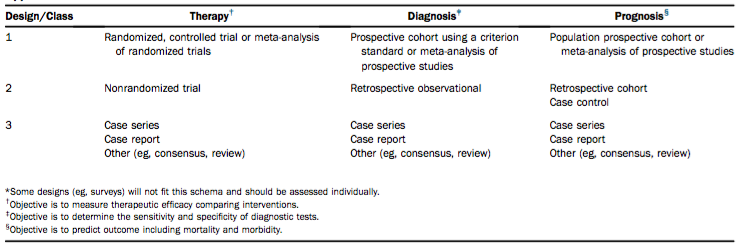
**

Supplement: S1 Table — (DOCX) [file pone.0178456.s003.docx]
